# From primary to secondary care level: Assessing patient retention of periodontal staging and grading information

**DOI:** 10.1002/jper.70008

**Published:** 2025-09-26

**Authors:** Pasquale Santamaria, Rohan Mangalpara, Thamara Kumar, Tina Lipovec, Luigi Nibali

**Affiliations:** ^1^ Periodontology Unit Centre for Host Microbiome Interactions Faculty of Dentistry Oral & Craniofacial Sciences King's College London London UK

**Keywords:** patient education, patient knowledge, periodontal grading, periodontal staging, periodontitis, secondary care

## Abstract

**Background:**

Accurate communication of periodontal stage and grade by general dentists in primary care is critical for patient understanding and engagement, yet patient retention and self‐reporting of this information upon referral to secondary care remains unclear.

**Methods:**

A total of 372 periodontal patients referred were informed about their diagnosis by their general dentists and then referred to secondary care level. Data were collected through an eight‐item periodontal staging and grading (PSG) questionnaire, along with demographic, medical, and dental records. Periodontal diagnoses were classified by a specialist using the 2018 classification system. Associations between clinical diagnosis and patient perception were analyzed using Chi‐square tests and Spearman's rank correlation.

**Results:**

While 46.9% of patients diagnosed with periodontitis reported to be informed of their condition, only 19.3% reported knowing their specific stage and grade. Among patients with advanced periodontitis (stage III/IV), self‐reported severity often aligned with clinical staging. However, for early‐stage disease (stage I/II), perceptions were less accurate, and only 30.2% of grade C patients recognized rapid progression. Significant correlations were found between patient‐reported symptoms and clinician‐assigned staging: tooth loss (*ρ* = 0.69, *p* < 0.0001), root exposure (*ρ* = 0.638, *p* < 0.0001), and tooth mobility (*ρ* = 0.55, *p* < 0.0001).

**Conclusion:**

Most patients referred to secondary care lacked information on their disease stage and grade. Severe stages and grades were better perceived by patients compared with mild forms of periodontitis. The PSG questionnaire offers a valuable tool for identifying knowledge gaps and guiding tailored discussions in clinical practice.

**Plain language summary:**

Our study asked 372 people referred for gum disease to fill out a simple eight‐question survey about how they see their own gum health and compared their answers to the detailed diagnosis made by periodontists. We found that fewer than one in five patients knew exactly how severe their disease was or how fast it was progressing. People with more advanced gum damage generally understood their condition, but those with mild disease often thought they were healthier than they really were. When patients reported losing teeth, seeing root surfaces, or noticing loose teeth, these experiences matched closely with the clinical measures of disease severity. These results show that many patients lack clear information about their gum health and that a brief questionnaire can help dentists pinpoint misunderstandings, improve patient education, and support better treatment decisions.

## 1 INTRODUCTION

The accurate definition of staging and grading of periodontitis is essential for the correct diagnosis and subsequent management of this chronic health concern worldwide.^1^ In fact, defining cases of periodontitis correctly can lead to better estimation of disease prevalence,^2^ improved screening of patients who need periodontal treatment,^3^ more accurate determination of prognosis,^4^ and the interpretation of research findings.^5^, ^6^ The 2018 classification system divides periodontitis into four stages (ranging from I to IV) and three grades (A, B, and C), providing a framework to evaluate the severity and complexity of the disease and identifying patients who are more likely to require greater effort to prevent or control their chronic disease over the long term.^3^

As the first point of contact for many patients with periodontitis, general dental practitioners (GDPs) play a crucial role in diagnosing and classifying the periodontal disease by assigning a stage and grade, which are essential for determining appropriate treatment strategies.^7^ The current diagnostic system involves the use of traditional measures such as probing pocket depth, clinical attachment loss, and bone loss to reach a final classification. However, as reported in previous studies, both international experts and general dentists demonstrated a higher accuracyin staging severe periodontitis, while their accuracy in assigning grading was moderate.^8^, ^9^

Successful treatment of periodontitis relies not only on the ability of the clinician^10^ but also on patients' compliance and their adherence to the treatment plan^11^. Therefore, it is well known that enhanced communication with the patient may lead to improved patient education, may offer a more comprehensive assessment of periodontal diseases, and ultimately support better adherence to treatment plans.^12^, ^13^ The use of patient‐reported outcomes (PROs)^14^ continues to grow beyond clinical research, recognizing their potential to transform health care and enhance quality and safety by placing patients at the center of decision‐making.^15^ Therefore, the implementation at patient level of how periodontitis is classified may help both patients and clinicians to optimize a tailored treatment plan, marking a significant step toward personalized care.^16^

Despite the new classification system of periodontitis being widely used, patient perceptions of disease severity (staging) and progression (grading) remain underexplored. Therefore, this study primarily aimed to assess the agreement between patient self‐reported stage and grade and the specialist's diagnosis at secondary‐care level among patients who report having been informed of their exact periodontal stage and grade by their general dentist. The secondary aim was to determine in patients who reported not having been informed of their exact stage and grade by their general dentist, whether they can nevertheless identify their specific stage and grade of periodontitis and report key clinical signs associated with their disease as gingival recessions, tooth mobility, tooth loss, and masticatory dysfunction.

## 2 MATERIALS AND METHODS

This survey evaluation included an integrated analysis of demographic, medical, and dental records and data collected from an eight‐item periodontal staging and grading questionnaire (PSG). The population consisted of a cohort of patients referred for periodontal reasons by general dental practitioners/other dental professionals to receive secondary care at the Periodontology Unit of the Guy's and St Thomas' Hospital, London (UK).

Eligible participants involved in King's College London Oral, Dental and Craniofacial Biobank were included in this study (National Health Service [NHS] UK Research Ethics Service approval reference 20/EE/0241, provided by the East of England‐Cambridge East Research Ethics Committee). Each participant provided written consent to enroll in the Biobank. Approval to access data and samples for the present study was obtained from the Biobank Management Committee (REF027). The safety of participants' data was maintained by following the current information governance regulations (GDPR). All participants' data were anonymized, encrypted, and physically stored in a protected data point. The survey was conducted for 14 months, from November 2023 to January 2025.

### 2.1 Guidelines for referral from primary to secondary care level

According to the NHS (UK) guidelines, these authors assumed that at primary care level, periodontal patients were firstly informed by the general dentist practitioners (GDPs) about their periodontal diagnosis, including the staging and grading of periodontitis. Patients diagnosed with periodontitis initially received periodontal treatment with their GPDs. If this was not successful enough to reach periodontal stability, the general dentist referred the patient to the Periodontology Unit of Guy's and St Thomas' Hospital, London (UK). The referral included: (a) periodontal diagnosis with six‐point pocket charts if the referral was for periodontal disease, (b) current diagnostic electronic radiographs of the affected sites clearly demonstrating periodontal bone levels, and (c) a plaque distribution chart and % plaque score.

### 2.2 Patient screening and assessment at secondary care level

Dental referrals were accepted/rejected at secondary care level based on the following acceptance criteria:

1. Periodontitis, such as stage III/IV, grade B/C in patients who had at least one course of oral hygiene instruction, supra‐gingival and sub‐gingival PMPR with local anesthesia with their general dentist.
2. Other plaque and non‐plaque induced periodontal disease.
3. Mucogingival problems, such as gingival recession.

Once the referral was accepted, patients were scheduled for an appointment to receive a full periodontal examination. As per secondary care level, the following steps were followed: (1) the GDP's written referral was reviewed before the periodontal examination including the reason for referral (periodontitis, other periodontal diseases), (2) collection of medical and dental history, (3) accurate patient‐related periodontal history, periodontal risk factor assessment and oral hygiene habits (e.g., familiarity, frequence of toothbrushing, interdental brushing, etc.), (4) extra‐ and intra‐oral examination (soft and hard tissues), (5) full mouth six‐point pocket chart examination, 6) radiological examination (periapical x‐rays, OPG, cone‐beam computed tomography) according to clinical need.

At the end of the periodontal examination, each case of periodontitis was accurately classified using the 2018 classification^1^ (staging, grading, and extension) by a consultant and UK specialist in periodontics. Clinical measurements were collected according to the Biobank standard operational protocol presented in a previous study.^17^

### 2.3 Questionnaire administration and analysis

At secondary care level, each participant was asked to fill out the PSG questionnaire before starting the full periodontal examination. A copy of the questionnaire can be found in Supplementary Material 1 in the online *Journal of Periodontology*. The first six items (from number 1 to 6) were revised on the basis of the validated CDC/AAP questionnaire for surveillance of periodontitis.^18^ The last two items (numbers 7 and 8) focused on assessing the patient‐reported knowledge of staging and grading of periodontitis.

The eight‐item questionnaire covered the following aspects:

1. Perception of periodontal status (health/disease);
2. Knowledge of periodontal diagnosis (stage and grade);
3. Knowledge/Perception of stage of periodontitis (mild, moderate, severe, very severe);
4. Knowledge /Perception of grade of periodontitis (slow, moderate, rapid);
5. Self‐reported signs of periodontitis (gingival recessions, tooth mobility, tooth loss, masticatory disfunction).

### 2.4 Sample size and data analysis

As no formal sample size calculation could be performed, due to the absence of previous similar studies, a convenience sample was included. The internal consistency of multi‐item scales was assessed using Cronbach's α, which estimates the degree to which responses to individual items correlate with one another and hence reflect a common underlying construct. Values of α range from 0 to 1, with higher values indicating greater reliability; in line with convention, α ≥ 0.70 was considered acceptable, α ≥ 0.80 good, and α ≥ 0.90 excellent. The coefficient was computed separately for each scale using SPSS (29.0.2.0^1^) reliability analysis module. Data from the questionnaires were analyzed descriptively. Further analysis was done by reporting responses separately by participants' group and by characteristics such as the awareness of gum disease (yes or no), patient‐reported staging and grading, and clinician‐reported staging and grading. Chi‐square test was carried out to assess differences in responses across these different groups of participants for categorical variables. To examine the association between patient‐reported symptoms and clinically determined periodontitis stage (an ordinal variable), it was calculated Spearman's rank‐order correlation coefficient (ρ). Each binary or ordinal symptom item (e.g., visible roots, tooth mobility, history of tooth loss, awareness of periodontal pockets, masticatory difficulty) was coded numerically and tested against the three‐level stage variable (II–IV). All correlations were two‐tailed, with a significance threshold set at *p* < 0.05. Analyses were conducted in SPSS (version 27; IBM Corp), using the “Correlations” module, and correlation matrices were visualized as a heatmap generated in R (version 4.1) via the ggplot2 and pheatmap packages.

## 3 RESULTS

A total of 372 participants were included in the analysis. Three participants withdrew from the biobank and were therefore not included (see Supplementary Material 2 in the online *Journal of Periodontology*). The mean age of the cohort was 49 years, and 60% of the participants were female. The cohort was composed of individuals identifying as White (59.78%), Afro‐Caribbean (17.96%), Asian (14.48%), Asian‐White (6.43%), and prefer not to say (1.34%). Although this was not a previously published, validated scale, it was tested for reliability (Cronbach's alpha). The result (α = 0.886) suggested optimal reliability of all items in the scale, and the decision to retain them all for analysis was made on this basis.

Most participants were diagnosed with periodontitis, distributed as follows: stage III (49.5%), stage IV (25%), stage II (9.9%), and stage I (2.2%). Regarding disease grading, 65.5% were classified as grade C, 16.3% as grade B, and 4% as grade A. A minority (13.4%) were not diagnosed with periodontitis (22 gingivitis, 28 healthy or with gingival recessions).

### 3.1 Correlation between staging and patient‐reported stage of severity

As shown in Table 1, among the 334 participants who responded to question 7, “What do you think the stage of severity of your gum disease is?,” perceptions of gum disease severity varied by stage. In stage III periodontitis (*n* = 184), 35.9% perceived their condition as severe, while 23.4% as a moderate stage. In stage IV (*n* = 93), 40.9% considered it severe, with 23.7% reporting a very severe stage. For stage II (*n* = 37), perceptions ranged from moderate (27%) to mild (21.6%). In stage I (*n* = 8), 50% rated it as moderate, while 37.5% considered it very severe. Among those without periodontitis (*n* = 50), 42% reported no gum disease, with others expressing varied levels of severity.

**TABLE 1:** Correlation between staging and patient‐reported stage of severity.

Q7 What do you think the stage of severity of your gum disease is?										
		Periodontal diagnosis stage								
		0	I	II	III	IV	Total	Chi‐square	df	*p*
1. Mild	Count	4	1	8	16	4	33	87,342	20	<0.001
	%	8.0%	12.5%	21.6%	8.7%	4.3%	8.9%			
2. Moderate	Count	8	4	10	43	14	79			
	%	16.0%	50.0%	27.0%	23.4%	15.1%	21.2%			
3. Severe	Count	6	0	4	66	38	114			
	%	12.0%	0.0%	10.8%	35.9%	40.9%	30.6%			
4. Very Severe	Count	2	3	3	20	22	50			
	%	4.0%	37.5%	8.1%	10.9%	23.7%	13.4%			
5. I do not have gum disease	Count	21	0	10	21	6	58			
	%	42.0%	0.0%	27.0%	11.4%	6.5%	15.6%			
6. Not answered	Count	9	0	2	18	9	38			
	%	18.0%	0.0%	5.4%	9.8%	9.7%	10.2%			
Total	Count	50	8	37	184	93	372			
	%	100.0%	100.0%	100.0%	100.0%	100.0%	100.0%			

### 3.2 Correlation between grading and patient‐reported rate of progression

As shown in Table 2, among the 325 participants who responded to question 8, “How rapidly do you think your gum disease is causing bone loss?,” perceptions of bone loss progression varied by diagnosis. In grade C periodontitis (*n* = 246), 24.8% perceived rapid progression, 36.6% moderate, and 15.9% slow, while 11.4% denied having gum disease. For grade B (*n* = 61), 41% perceived moderate progression, 16.4% rapid, and 18% slow, with 14.8% stating they had no gum disease. In grade A (*n* = 15), 20% reported moderate progression, 6.7% rapid, and 13.3% slow, while 33.3% denied having gum disease. Among those without periodontitis (*n* = 50), 44% stated they had no gum disease, with others reporting varied perceptions of rapidity of progression.

**TABLE 2:** Correlation between grading and patient‐reported grade of severity.

Q8 How rapidly do you think your gum disease is causing bone loss?									
		Periodontal diagnosis grade							
		0	A	B	C	Total	Chi‐Square	df	*p*
1. Slow rate	Count	8	2	11	39	60	57,135	16	<0.001
	%	16.0%	13.3%	18.0%	15.9%	16.1%			
2. Moderate rate	Count	7	3	25	90	125			
	%	14.0%	20.0%	41.0%	36.6%	33.6%			
3. Rapid rate	Count	4	1	10	61	76			
	%	8.0%	6.7%	16.4%	24.8%	20.4%			
4. I do not have gum disease	Count	22	5	9	28	64			
	%	44.0%	33.3%	14.8%	11.4%	17.2%			
5. Not answered	Count	9	4	6	28	47			
	%	18.0%	26.7%	9.8%	11.4%	12.7%			
Total	Count	50	15	61	246	372			
	%	100.0%	100.0%	100.0%	100.0%	100.0%			

### 3.3 Patient knowledge of diagnosis of periodontitis

As shown in Table 3, a total of 372 participants responded to the question: “Have you ever been told by a dentist that you have gum disease with bone loss?” Among those diagnosed with periodontitis (*n* = 305), 46.9% reported being informed of their condition but were unaware of its stage and grade, while 19.3% were both aware of their diagnosis and its severity. Additionally, 25.5% reported never being diagnosed with gum disease, and 8.4% did not answer.

**TABLE 3:** Patient knowledge of diagnosis of periodontitis. Participants' answers to the question: “Have you ever been told by a dentist that you have gum disease with bone loss?”

Q1 Have you ever been told by a dentist that you have gum disease with bone loss?								
		Health	Gingivitis	Periodontitis	Total	Chi‐Square	df	*p*
(A) Yes, but not aware of stage and grade	Count	8	6	151	165	44.54	6	<0.001
	%	28.6%	27.3%	46.9%	44.4%			
(B) Yes, and aware of stage and grade	Count	2	3	62	67			
	%	7.1%	13.6%	19.3%	18.0%			
(C) No	Count	10	9	82	101			
	%	35.7%	40.9%	25.5%	27.2%			
Not answered	Count	8	4	27	39			
	%	28.6%	18.2%	8.4%	10.5%			
Total	Count	28	22	322	372			
	%	100.0%	100.0%	100.0%	100.0%			

Among participants diagnosed with gingivitis, 40.9% reported never being told they had gum disease with bone loss but approximately 40% reported they had been told to have periodontitis.

### 3.4 Patient‐reported stage of disease in relation to periodontitis grade

**FIGURE 1:**
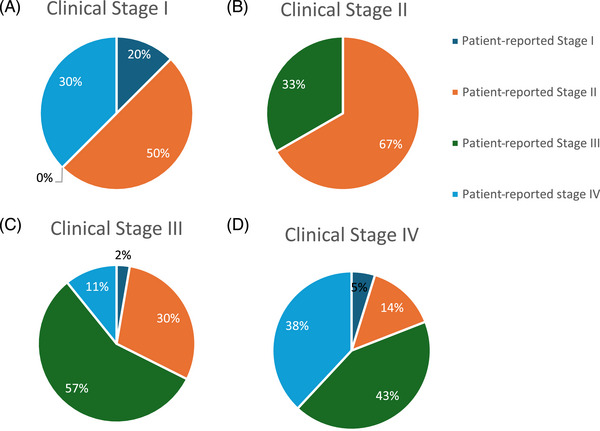
Patient‐reported severity of disease in relation to periodontitis stage.

Among the 67 participants who reported being informed of both the stage and grade of their periodontitis, an analysis was conducted to compare their diagnosed stage with their self‐reported perception of severity (Figure 1 A–D). The majority of participants diagnosed with stage III periodontitis (*n* = 37) perceived their condition as stage III (56.8%), while 29.7% identified it as stage II, 10.8% as stage IV, and 2.7% as stage I (Figure 1A). Similarly, among those diagnosed with stage IV periodontitis (*n* = 21), 42.9% classified their disease as stage III, 38.1% as stage IV, 14.3% as stage II, and 4.8% as stage I (Figure 1B). For participants diagnosed with stage II periodontitis (*n* = 3), 66.7% perceived their disease as stage II, while 33.3% classified it as stage III (Figure 1C). Only one participant diagnosed with stage I periodontitis (*n* = 1) identified their condition as very severe.

### 3.5 Patient‐reported rate of disease progression in relation to periodontitis grade

**FIGURE 2:**
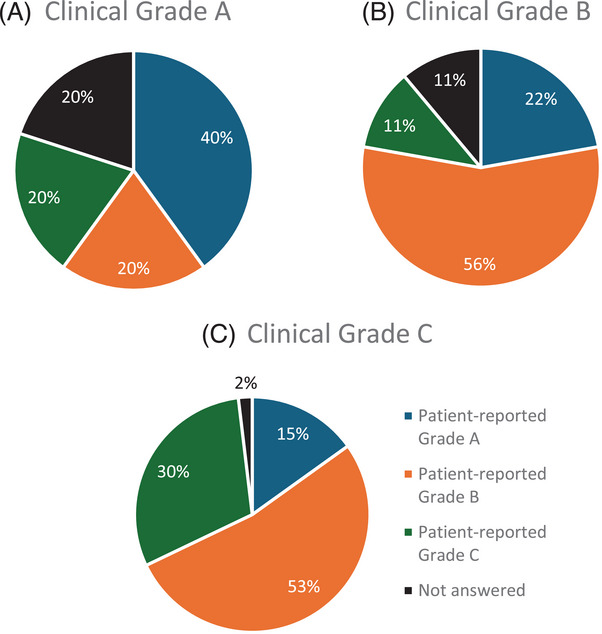
Patient‐reported rate of disease progression in relation to periodontitis grade.

Figure 2A–C shows the distribution of the 67 participants who reported being aware of their periodontitis grade based on their diagnosed periodontitis grade. For individuals diagnosed with grade C periodontitis (n = 53), the majority (52.8%) reported their bone loss as occurring at a moderate rate, while 30.2% stated it was rapid, and 15.1% considered it slow. Only 1.9% did not provide an answer (Figure 2A). Among those diagnosed with grade B periodontitis (n = 9), 55.6% reported a rapid rate of bone loss, 22.2% as slow, and 11.1% considered it moderate (Figure 2B). For participants diagnosed with grade A periodontitis (*n* = 5), 40% reported a slow rate of bone loss, while 20% each classified it as moderate, rapid, or not applicable (Figure 2c).

### 3.6 Self‐identification of periodontitis severity among uninformed participants

**FIGURE 3:**
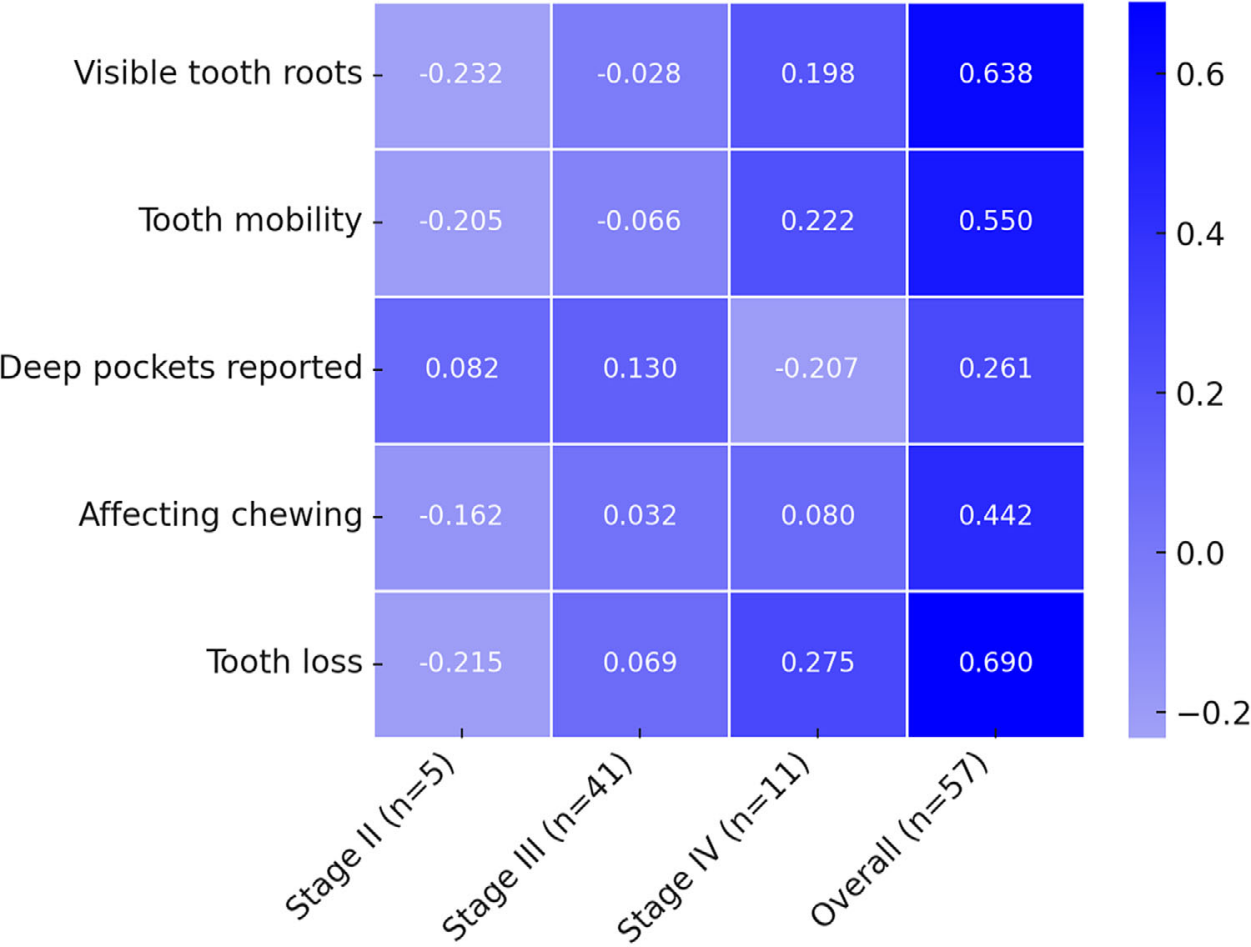
Spearman's rank correlation between patient‐reported symptoms and periodontitis.

Among the 165 participants with periodontitis who reported being uninformed of their periodontitis stage and grade, an analysis was conducted to assess whether they could still accurately identify their disease severity based on a set of diagnostic questions. A heatmap was generated to illustrate the correlation between participants' responses and their actual diagnoses (Figure 3). A total of 57 individuals were able to identify the exact stage of their disease (41 participants with stage III, 11 with stage IV, and 5 with stage II). All individuals in stage IV (100%), 85.4% in stage III, and 60% replied they could see more of their roots. A total of 90.9% of stage IV, 68.3% of stage III, and 40% of stage II reported tooth hypermobility. All stage II participants reported that they did not lose teeth for gum disease, 72.7% of stage IV reported they lost more than 4 teeth, in stage III, 46% reported they lost <4 teeth, and 41.5% reported no tooth loss. In terms of masticatory difficulty, 81.8% of stage IV and 63.4% of stage III reported that gum disease was affecting their bite. Sixty percent of stage II cases reported that no one mentioned having deep pockets, while 81.8% of stage IV and 70.7% reported yes to the same question.

Spearman's Rank Correlation was used to investigate the correlation between patient‐reported symptoms and periodontitis stage (Figure 3). Statistically significant positive correlations were observed for all questions. Specifically, “tooth loss due to gum disease” showed the strongest correlation (*ρ* = 0.69, *p* < 0.0001), followed by “visible tooth roots” (*ρ* = 0.638, *p* < 0.0001) and “tooth mobility” (*ρ* = 0.55, *p* < 0.0001). “Being told about deep pockets” exhibited a weak positive correlation (*ρ* = 0.261, *p* = 0.0001), as did “bite affected” (*ρ* = 0.442, *p* = 0.003).

## 4 DISCUSSION

This study aimed to explore the correlation between clinician‐diagnosed staging and grading of periodontitis and the self‐reported disease severity and progression among patients referred from primary care to secondary care using an eight‐item questionnaire. These findings underscore the significant variability in patient knowledge of their condition in terms of the stage and grade of their periodontitis, and the challenges faced in achieving alignment between patient perceptions and clinical diagnoses. This study revealed that only 20% of patients with stage III‐IV periodontitis were able to report their diagnosis. Almost half of participants diagnosed with periodontitis were informed of their condition but lacked knowledge of its stage and grade, which may potentially contribute to reduced patient engagement with treatment strategies. This aligns with previous research indicating that individuals with periodontitis often exhibit insufficient understanding of their condition, coupled with negative attitudes and passive behaviors toward disease management.^19^ Enhancing patient education could play a critical role in improving their knowledge, which in turn may lead to more proactive attitudes and better engagement with recommended treatment and self‐care practices.^19^ These findings emphasize the importance of improving patient education to bridge the knowledge gap about periodontitis severity and progression, particularly the need for better communication regarding the staging and grading system used by clinicians.^20^

Considering the subset of patient who reported being told by a dentist of their periodontal stage and grade, the correlation between clinician‐diagnosed stage and patient‐reported perceptions of disease severity was fairly consistent. Patients in stage III and IV perceived their condition as severe or very severe, which aligns with clinical expectations given the advanced nature of stage III/IV periodontitis. On the other hand, when analyzing perceptions of disease progression, a variable trend emerged where more than half of participants diagnosed with grade C periodontitis, the most severe grade, perceived their bone loss progression as moderate. The results suggest that patient perceptions of disease progression rate align more closely with clinical grading for moderate forms of the disease, but less so for those with severe forms. This highlights that patient knowledge is often influenced more by signs/symptoms like tooth sensitivity or toothache, rather than strictly by clinical measures of attachment loss or bone resorption. This finding suggests the need for improved methods of educating patients about the implications of their grading, ensuring they understand not only their diagnosis but also the long‐term consequences of untreated or poorly managed periodontitis. Another aspect that should be taken into consideration is the patient's understanding of staging and grading. Our questionnaire deliberately avoided such technical terms, instead using plain language descriptors like “severity” and “rate of progression” of gum disease, which are more accessible and meaningful to patients in routine care settings. Furthermore, it is important to consider that the accuracy of the staging and grading initially communicated to the patient may not have been entirely reproducible. Marini et al. conducted a study to evaluate the accuracy of periodontitis staging and grading by general dentists, finding that staging accuracy was higher than grading accuracy.^8^ Our results indirectly support these findings, as we observed that periodontal patients who were informed by their general dentist about both their stage and grade were more likely to correctly identify their stage rather than their grade.

Interestingly, in line with previous research^5^ a considerable proportion of participants who were unaware of their disease stage still displayed an understanding of some of the disease's characteristics, such as tooth loss, tooth mobility, root exposure, that helped them to correctly identify the actual periodontitis staging. The analysis showed that patients with self‐reported signs of tooth loss due to gum disease, root visibility, and tooth mobility showed significant associations with the self‐diagnosed stage of periodontitis. Being told about deep pockets and chewing dysfunction were found to have weaker correlations, suggesting that while these factors may contribute to patients' understanding of their disease, they are less directly linked to accurate self‐assessment of disease severity. Interestingly, these findings suggest that higher disease's sign and symptoms severity is associated with the patient ability to correctly identify more advanced periodontitis stages (stage III/IV), with “tooth loss” being the strongest indicator.

The results of this study have several important implications for clinical practice. Firstly, these findings suggest an opportunity to improve periodontal care standards in the UK by strengthening communication between clinicians and patients. The PSG questionnaire developed in this study could serve as a practical tool to support both patient education and training of general dental practitioners (GDPs) in effectively conveying staging and grading concepts. University units could lead in disseminating such tools, while organizations like the British Society of Periodontology and Implant Dentistry (BSP) and NHS might consider integrating structured communication strategies into routine care to promote more informed, person‐centered treatment. By evaluating patients' comprehension of their periodontal condition, the questionnaire can help identify gaps in knowledge that may hinder effective disease management.^21^ Furthermore, this tool can be valuable beyond initial diagnosis, serving as a means to monitor patients' symptoms and perceptions of their disease over time.^22^ Regular use of this questionnaire may facilitate early identification of concerns or misconceptions, allowing for timely interventions that improve patient engagement, treatment adherence, and overall periodontal health outcomes.^12^ Moreover, clinicians should recognize that while patients may be unaware of specific clinical stages and grades, they are often able to report symptoms that correlate with more advanced disease, such as tooth mobility, tooth loss, and visible roots. Thus, clinicians should not only rely on clinical measures but also consider the patient perspective when determining treatment plans.^5^

While this study provides valuable insights into the relationship between clinical staging and grading and patient perceptions, there are several limitations. First, these findings should be interpreted within the context of the NHS referral system and academic hospital setting, where patients may receive more time‐intensive care and education. As patients were not always informed using specific terms like “staging” and “grading,” and given the structured nature of the UK system, results may not be generalizable to private or international settings with different resources or communication practices. Additionally, the self‐reported nature of the data may introduce bias, as patients may have been told about staging and grading by a variety of clinicians (GDPs, hygienists, other specialists). In addition, patient may perceive their disease differently based on their subjective experience or memory recall. Furthermore, the study did not assess how the education level or socio‐economic status of participants influenced their understanding of periodontitis, which may be an important factor in shaping patient knowledge. Future studies could expand on this research by including a more diverse population, including individuals from primary care settings, and exploring the impact of different educational interventions on patient knowledge and disease management. Longitudinal studies could also help clarify the relationship between patient understanding and long‐term disease outcomes.

## 5 CONCLUSION

In conclusion, this study highlighted the significant gap between clinician‐diagnosed staging and grading of periodontitis and patient self‐awareness of disease severity and progression. The results suggested that while patients with advanced stages of periodontitis often perceive their condition correctly, those with milder forms of the disease may struggle to understand its severity. Although our study did not directly assess treatment engagement or patient attitudes, improved communication may contribute to enhanced adherence and outcomes. Future research should investigate whether patient knowledge of periodontal status influences their motivation, expectations, and commitment to treatment.

## AUTHOR CONTRIBUTIONS

Pasquale Santamaria was involved in study conception, analysis, and manuscript drafting. Pasquale Santamaria and Luigi Nibali were involved in study design. Rohan Mangalpara, Thamara Kumar, Tina Lipovec, and Luigi Nibali were involved in data collection. All authors have been involved in data interpretation and revising the manuscript critically and have given final approval of the version to be published.

## CONFLICT OF INTEREST STATEMENT

The authors report no conflicts of interest related to this study.

## FUNDING INFORMATION

This study was not funded.

## Supporting information



Supporting Information

Supporting Information

## Data Availability

The datasets generated during and/or analyzed during the current study are available from the corresponding author upon reasonable request.
