# Supplementary material for: From primary to secondary care level: Assessing patient retention of periodontal staging and grading information
Source: J Periodontol. 2025 Sep 26;97(2):326–35. doi: 10.1002/jper.70008 (PMC13001134; doi:10.1002/jper.70008)
Supplement: Supplementary file 1 — Supporting Information [file JPER-97-326-s001.docx]

**
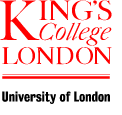

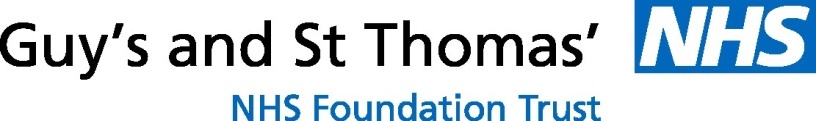
**

Version 1.0, 12 September 2023

PERIO 10-009

IRAS project ID:255079

Participant Biobank Number _____________

**PERIODONTAL CLASSIFICATION for participants in Dental, Oral and Craniofacial Biobank**

1. Have you ever been told by a dentist that you have gum disease with bone loss?

1. Yes, but I do not know what the stage of severity and grade of progression of my gum disease is.
2. Yes, I also know the exact stage of severity and grade of progression of my gum disease.
3. No.

2.  Do you think you can see more of roots of teeth than in past?

1. Yes
2. No

3. Have you noticed that some of your own teeth have come loose or fallen out on their own?

1. Yes
2. No

4. Have you lost teeth due to gum disease, or because they were loose/wobbly?

1. Yes, more than 4 teeth.
2. Yes, but no more than 4 teeth.
3. No

5. Have you been told you have deep pockets?

1. Yes
2. No

6. Do you think your gum disease is affecting your bite, or your chewing ability?

1. Yes
2. No

7. What do you think the stage of severity of your gum disease is?

1. Mild
2. Moderate
3. Severe
4. Very severe
5. I do not have gum disease

8. How rapid do you think your gum disease is causing bone loss?

1. slow progression
2. moderate progression
3. rapid progression
4. I do not have gum disease
