# Supplementary material for: From primary to secondary care level: Assessing patient retention of periodontal staging and grading information
Source: J Periodontol. 2025 Sep 26;97(2):326–35. doi: 10.1002/jper.70008 (PMC13001134; doi:10.1002/jper.70008)
Supplement: Supplementary file 2 — Supporting Information [file JPER-97-326-s002.docx]

Table 1

| **Patient-level** | **Average** |
| --- | --- |
| **Participants included** | 372 |
| **Age (mean, SD)** | 49.85 (15.75) |
| **Biological Sex** | **Frequency (%)** |
| **Male** | 150 (40%) |
| **Female** | 225 (60%) |
| **Ethnicity** | **Frequency (%)** |
| **Caucasian** | 223 (59.78%) |
| **Afro-Caribbean** | 67 (17.96%) |
| **Asian** | 54 (14.48%) |
| **Other ethnicity** | 24 (6.43%), |
| **Prefer not to say** | 5 (1.34%). |
| **Periodontal Diagnosis** | **Frequency (%)** |
| **Healthy/Gingivitis/Other Diagnosis** | 50 (13.4%) |
| **Periodontitis Stage I** | 8 (2.2%) |
| **Periodontitis Stage II** | 37 (9.9%) |
| **Periodontitis Stage III** | 184 (49,5%) |
| **Periodontitis Stage IV** | 93 (25%) |
| **Periodontitis Grade A** | 15 (4%) |
| **Periodontitis Grade B** | 61 (16,3) |
| **Periodontitis Grade C** | 246 (65,6%) |
